# Transparency of informed consent in pilot and feasibility studies is inadequate: a single-center quality assurance study

**DOI:** 10.1186/s40814-021-00828-w

**Published:** 2021-04-16

**Authors:** Mohammed I.U. Khan, Lawrence Mbuagbaw, Matthew Holek, Faris Bdair, Zoha H. Durrani, Katie Mellor, Saskia Eddy, Sandra M. Eldridge, Claire L. Chan, Michael J. Campbell, Christine M. Bond, Sally Hopewell, Gillian A. Lancaster, Lehana Thabane

**Affiliations:** 1 Biostatistics Unit, St. Joseph’s Healthcare, Hamilton, Ontario Canada; 2 University of Toronto, Toronto, Ontario Canada; 3 Department of Health Research Methods, Evidence and Impact, McMaster University, 1280 Main Street W, Hamilton, ON L8S 4L8 Canada; 4 Joan C. Edwards School of Medicine, Huntington, WV USA; 5 Oxford Clinical Trials Research Unit/Centre for Statistics in Medicine, University of Oxford, Oxford, UK; 6 Institute of Population Health Sciences, Barts and the London School of Medicine and Dentistry, Queen Mary University of London, London, UK; 7 School of Health and Related Research, University of Sheffield, Sheffield, South Yorkshire UK; 8 Institute of Applied Health Sciences, University of Aberdeen, Aberdeen, Scotland; 9 School of Medicine, Keele University, Newcastle under Lyme, UK

**Keywords:** Research ethics, Informed consent, Pilot studies, Feasibility studies

## Abstract

**Background:**

Pilot and feasibility studies (PAFS) often have complex objectives aimed at assessing feasibility of conducting a larger study. These may not be clear to participants in pilot studies.

**Methods:**

Here, we aimed to assess the transparency of informed consent in PAFS by investigating whether researchers communicate, through patient information leaflets and consent forms, key features of the studies. We collected this data from original versions of these documents submitted for ethics approval and the final approved documents for PAFS submitted to the Hamilton Integrated Research Ethics Board, Canada.

**Results:**

One hundred eighty-four PAFS, submitted for ethics approval from 2004 to 2020, were included, and we found that of the approved consent documents which were provided to participants, 83.2% (153) stated the terms “pilot” or “feasibility” in their title, 12% (22) stated the definition of a pilot/feasibility study, 42.4% (78) of the studies stated their intent to assess feasibility, 19.6% (36) stated the specific feasibility objectives, 1.6% (3) stated the criteria for success of the pilot study, and 0.5% (1) stated all five of these criteria. After ethics review, a small increase in transparency occurred, ranging from 1.6 to 2.8% depending on the criteria. By extracting data from the protocols of the PAFS, we found that 73.9% (136) stated intent to assess feasibility, 71.2% (131) stated specific feasibility objectives, and 33.7% (62) stated criteria for success of the study to lead to a larger study.

**Conclusion:**

The transparency of informed consent in PAFS is inadequate and needs to be specifically addressed by research ethics guidelines. Research ethics boards and researchers ought to be made aware and mindful of best practices of informed consent in the context of PAFS.

**Supplementary Information:**

The online version contains supplementary material available at 10.1186/s40814-021-00828-w.

## Background

A feasibility study is a preliminary investigation conducted with the purpose of assessing the feasibility of conducting a future larger study [1]. Pilot studies are a subset of feasibility studies, in which the intervention tested in the subsequent larger study is implemented, or partially implemented, on a smaller scale to assess feasibility [2]. Research guidance and researchers have been found to use the terms “pilot” and “feasibility” synonymously [3, 4], and because our study concerns all feasibility studies, we make no distinction between pilot and feasibility studies (PAFS) in our investigation.

For the purposes of our study, we identified three main reasons for conducting PAFS, based on the work of Thabane et al. [5], PAFS seek to answer feasibility questions related to process, resources, and management issues [5–7]. Process-related objectives seek to assess whether steps that need to be taken to ensure the success of the main study are feasible [5–7]. This includes assessing recruitment rates, acceptability of the intervention, loss to follow-up, and data collection tools. Resource-related objectives seek to assess whether issues of resources, like time, money, and capacity, could arise in the larger study [5–7]. Management-related objectives seek to assess whether issues of human and data management could arise in the subsequent study, including identifying challenges related to data storage and transfer [5–7]. Other frameworks for pilot studies include another category of objectives related to scientific aims, such as assessing efficacy and safety of interventions [5]. We consider these to be proof of concept or exploratory studies and thereby do not include them in our definition of PAFS.

However, ethical issues of informed consent in PAFS remain largely unaddressed, as they are hardly discussed in the literature surrounding informed consent and PAFS [5]. Specifically, the obligation of researchers to communicate the feasibility nature and objectives of PAFS to participants of these studies, when obtaining consent, is an important ethical concern that has not yet been appropriately addressed by research ethics guidelines or investigated by researchers.

The objectives of PAFS can be less intuitive, and from participants’ perspectives, the studies’ objectives may appear to be the same as large scale trials and studies. Since informed consent in PAFS is not addressed in many popular research ethics guidelines, [5] there is substantive concern that informed consent in PAFS lacks transparency [5, 8]. Transparent communication between researchers and participants is essential to maintaining the rights of participants, credibility of researchers, and trust between researchers and the public. This is specifically relevant to PAFS, as many of them do not proceed to larger studies. If this is not communicated to participants, it can result in a unique form of therepeutic misconception—where the intended value of the research differs from the participant’s percieved value [8]. Additionally, if the feasibility nature of PAFS is not communicated, the quality of consent can be diminished by therepeutic misestimation—the tendency for potential participants to overestimate the benefits and underestimate the risks of participating in a study [8]. This is especially concerning in PAFS since their clinical benefits are reaped after completion of the main study, often much later; or if the pilot does not lead to a larger study there may be no clinical benefit. Only about 50% of published pilot studies stated a larger study was needed [9], and between 5 and 24% of pilot studies have been found to lead to future studies [3, 10], highlighting the importance of transparency during the informed consent process in PAFS.

Research ethics guidelines discuss informed consent as being well informed, meaning participants understand the nature, duration, purpose, methods, risks, discomforts, benefits, sources of funding, institutional affiliations of researchers, and potential conflicts of interest associated with the study [11–15]. Participants should also be informed of alternatives to participating in the study and have the opportunity to ask questions [11–15]. These principles apply to almost all studies involving participants, including pilot studies, with few exceptions, like when a waiver of consent or deception is required, as approved by a research ethics board (REB).

However, many research ethics guidelines, including the Nuremburg Code [11], Declaration of Helsinki [12], the Belmont Report [13], International Conference on Harmonization Good Clinical Practice [14], and the International Ethical Guidelines for Health-related Research Involving Humans [15] do not comment on informed consent in PAFS. The Tri-Council Policy Statement 2 (TCPS2) [16], Canada’s benchmark for the ethical conduct of research involving humans, is required to be followed in order to be eligible to receive government funding and was revised, in 2018, to include informed consent practices in pilot studies. Thabane et al. [5] have also developed recommendations for informed consent practices in pilot studies, but other research ethics guidelines need to be updated to address informed consent in PAFS, as evidence suggests, the reporting and methods are inadequately approached [3, 5, 9, 10].

Currently, it is unclear whether researchers are effectively communicating the feasibility nature of PAFS to their participants and providing acceptable levels of transparency in the informed consent process. After conducting an informal review of the literature for studies assessing practices of informed consent in PAFS, we concluded that this issue has not been empirically investigated in the literature. Since depriving participants of complete information on the PAFS they participate in has ethical implications and practical consequences, it is imperative that the issues of informed consent in PAFS are investigated urgently.

We hypothesized that a large number of PAFS approved for ethics clearance do not have a transparent informed consent process. This study is the first attempt to empirically investigate the transparency of informed consent in PAFS. We hope that by quantifying the severity of this issue at one center, we can begin to address it on a broader scale.

## Methods

Herein a brief overview of the methods is presented. Further details can be found in the published protocol by Khan et al. [17].

### Study objectives

Our primary objective was to assess whether PAFS submitted to the Hamilton integrated Research Ethics Board (HiREB), Canada, transparently communicate the purpose of the study to participants through their informed consent practice. The HiREB is McMaster University’s primary research ethics board. McMaster University is Canada’s most research-intensive, medical doctoral university [18], so the HiREB reviews many clinically oriented studies as well as studies from other departments at the university—including non-medical studies. For our purposes, a highly transparent informed consent practice requires that the informed consent documents, consisting of consent forms and participant information leaflets, effectively communicate: (1) the term “pilot” or “feasibility” in the title of the study; (2) the definition of a pilot or feasibility study; (3) the objectives or purpose of the study are stated clearly as assessing feasibility; (4) the specific feasibility objectives of the study; and (5) the progression criteria—the criteria for the feasibility study to successfully lead to the main study.

Our secondary objectives were (1) to assess whether there was a difference between the originally submitted informed consent documents and the final informed consent documents revised by the HiREB (revisions made in order to obtain research ethics approval), specifically in addressing the issues and criterion discussed in the primary objective; (2) to determine methodological characteristics associated with increased reporting or inclusion of the criterion discussed in the primary objectives within the original and revised informed consent documents; and (3) to assess the consistency with which PAFS assess feasibility outcomes as their primary objectives.

### Sample selection and size

All pilot and feasibility studies submitted to the HiREB, from January 2004 to December 2020, inclusive, that used the terms “pilot” or “feasibility” in their title and obtained participant consent were included in our study. All pilot and feasibility studies that had a waiver of consent were excluded.

To determine the sample size, we used the estimation method for a single proportion [17, 19]. The statistical formula behind the method uses an estimation for a single proportion—the proportion can be from a variable tested in the study and calculates the sample size needed for a given margin of error of the estimate and confidence interval (CI). In this method, an estimated proportion of 50% yields the largest necessary sample size. Thus, if the true proportion of pilot studies that use the term “pilot” or “feasibility” in the title of the study, as stated on the consent form, is 0.5, which will be estimated with a 95% CI and margin of error 0.40 to 0.60, then a sample size of 96 would be sufficient to address our objectives. We decided that if more than 96 studies met the inclusion criteria, we would include all of them in our study up to a maximum of 500, to decrease the width of the CI.

### Data collection

Anonymized data was extracted from the online HiREB database, with a subset of data (about 15%) extracted in duplicate. All disagreements in data collection were resolved by a third party, with a Kappa value and 95% CI calculated to assess agreement. Specific information collected from originally submitted and revised informed consent documents to address the primary objective and to compare transparency of original and revised documents included (1) if the term “pilot” or “feasibility” was in the title of the consent documents; (2) if a definition of pilot or feasibility study was stated; (3) if the objectives or purpose of the study was stated as to assess feasibility; (4) if the specific feasibility objectives of the study were stated; and (5) whether the progression criteria was stated.

Data extracted from the study protocols to address the secondary objectives included the following: use of randomization; whether the study was observational or interventional; whether data collected was quantitative, qualitative, or both; desired sample size; year of submission for ethics review; sources of funding; whether the study was approved by the REB; whether the study was labelled a “pilot” or “feasibility” study; whether intent to assess feasibility was stated; whether specific feasibility objectives were stated; whether progression criteria were stated; and the specific feasibility objectives of the studies.

### Data analysis

Data addressing the primary objective was used to calculate proportions of studies in the sample, with their corresponding 95% CI, that reported each item (from the primary objective), all five items, and at least one of the five items before and after HiREB review. Descriptive statistics were used to summarize characteristics of the studies. An exploratory analysis using multivariable binary logistic regression was used to determine characteristics associated with transparent consent practices, with the criteria from the primary objective used as the dependent variable and the independent variables being the study characteristics, including year of submission (before 2017 or after/during 2017); whether it was titled a pilot or feasibility study; study design (randomized, non-randomized with an intervention, or observational); type of data collected (quantitative, qualitative, or both); funding (industry sponsored or not), and whether the protocol stated progression criteria for the study to lead to a larger study. The Consolidated Standards of Reporting Trials (CONSORT) extension [20] for pilot studies was published in late 2016 and for this reason we categorized the study dates to before 2017 and after or during 2017. Multivariable binary logistic regression results are reported as odds ratios with 95% CIs used to assess statistical significance. Analysis was performed using IBM SPSS Statistics version 26.

### Ethics approval

This study was approved by the HiREB (project # 7071-C), which granted a waiver of consent for the research team to access the files and consent forms from pilot and feasibility studies submitted to the HiREB.

## Results

### Overview of studies

**Fig. 1:**
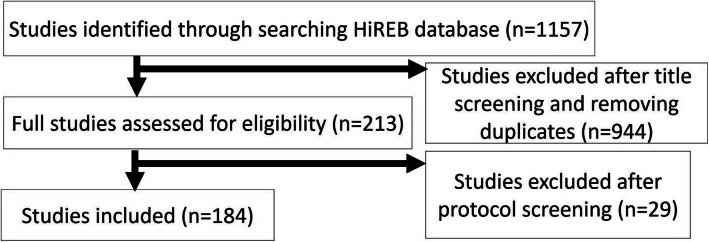
Flow diagram of the study inclusion process

After an initial search of the HiREB databases, 1157 studies were identified as potential PAFS. After removing duplicates and performing both title and full-text screenings, 184 studies were deemed eligible and included (Fig. 1). The studies included were conducted in Canada and submitted to the HiREB between 2004 and March of 2020. Most of the studies included were labelled as pilot studies, collected only quantitative data, were non-industry funded, and were submitted prior to 2017. Detailed descriptions of characteristics of studies included can be found in Table 1.

**Table 1:** characteristics of studies included (*n* = 184)

Study characteristic	n, (%)
Term used in title of the study	
“Pilot”	147, (79.9)
“Feasibility”	32, (17.4)
Both	5, (2.7)
Study design	
Randomized	63, (34.2)
Non-randomized interventional	47, (25.5)
Observational	74, (40.2)
Data collected	
Quantitative	122, (66.0)
Qualitative	12, (6.5)
Both	51, (27.6)
Year of submission	
2004-2016	135 (73.4)
2017 to 2020	49 (26.6)
Desired sample size	Median (min, max) 50 (5, 1152)
Industry funded	13, (7.1)

### Transparency of informed consent documents

Two hundred eighty observations from 30 studies were recorded in duplicate and a kappa value of 0.79 (95% CI 0.71, 0.83) was calculated for inter-rater reliability. Of the original informed consent documents submitted for ethics approval, 80.4% (95% CI 74.7, 86.2) included the terms “pilot” or “feasibility” in their titles; 9.2% (95% CI 5.1, 13.4) stated the definition of a pilot/feasibility study; 40.8% (95% CI 33.7, 47.9) stated the objectives of the study related to assessing feasibility; 19.6% (95% CI 13.8, 25.3) stated the specific feasibility objectives of the study; 1.6% (95% CI 0.0, 3.5) stated the progression criteria for the study (Table 2). Of the original informed consent documents submitted for ethics approval, 87.5% (95% CI 82.7, 92.3) of studies included at least one of the five criteria for transparency and only one study stated all five criteria for transparency (Table 2).

**Table 2:** Percentage of informed consent forms clearly communicating each of the criteria for transparency before and after research ethics board review (*n* = 184)

Item	Percentage of originally submitted studies with consent forms including the criteria (%) (95% confidence interval)	Percent of revised studies with consent forms including the criteria (%) (95% confidence interval)
“Pilot/feasibility” in title	80.4 (74.7, 86.2)	83.2 (77.7, 88.6)
Definition of pilot/feasibility study	9.2 (5.1, 13.4)	12.0 (7.3, 16.6)
Objectives state assessing feasibility	40.8 (33.7, 47.9)	42.4 (35.3, 49.5)
Specific feasibility objectives	19.6 (13.8, 25.3)	19.6 (13.8, 25.3)
Progression criteria	1.6 (0.0, 3.5)	1.6 (0.0, 3.5)
All five items above	0.5 (0.0, 1.6)	0.5 (0.0, 1.6)
At least one item	87.5 (82.7, 92.3)	89.7 (85.3, 94.1)

**Fig. 2:**
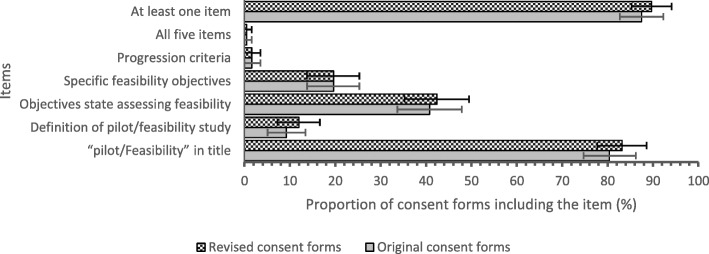
Percentage of informed consent forms clearly communicating each of the criteria for transparency before and after research ethics board review (*n* = 184). Error bars show 95% confidence intervals

After the informed consent documents were reviewed, revised, and approved by the HiREB, slight increases in the inclusion of criteria for transparency of informed consent occurred. Reporting of the terms “pilot” or “feasibility” in the titles of informed consent documents, stating the definition of a pilot/feasibility study, stating intent to assess feasibility, and studies including at least one item increased between 1.6 and 2.8% (Table 2). However, there was no increase in studies describing their specific feasibility objectives or progression criteria in their informed consent documents (Fig. 2).

### Pilot and feasibility study objectives according to study protocol

73.9% (95% CI 67.6, 80.3) and 71.2% (95% CI 64.7, 77.7) of PAFS had the aim of assessing feasibility and stated their specific feasibility objectives, respectively (Additional file 1: Appendix 1A). However, only about a third of studies stated their progression criteria (Additional file 1: Appendix 1A).

We found that 70.7% (95% CI 64.1, 77.2) of studies assessed process related feasibility objectives; 21.2% (95% CI 15.3, 27.1) assessed resource-related objectives; and 9.2% (95% CI 5.1, 13.4) assessed management-related feasibility objectives (Additional file 1: Appendix 1B). If a study stated any specific feasibility objectives, each objective was grouped into one of these three categories (process, resource, or management). Thus, almost a third of studies stated no specific feasibility objectives at all (Additional file 1: Appendix 1B).

### Characteristics of studies with transparent informed consent

Of the studies that incorporated the criteria for transparency in their originally submitted informed consent documents, most of them were labelled pilot studies, collected only quantitative data, were non-industry funded, submitted prior to 2017 and their protocols stated intent to assess feasibility and specific feasibility objectives (Additional file 2: Appendix 2). Moreover, most of the studies that stated specific feasibility objectives in their informed consent documents were observational studies. The same trends were found after REB review (Additional file 2: Appendix 2). The fifth criteria—whether the studies stated the progression criteria for the study to lead to a larger study in the informed consent documents—was not included in the tables since only three studies reported this item.

Looking at the relative inclusion of the criteria for transparency between studies of differing characteristics, in the originally submitted informed consent documents, we found that studies labelled “feasibility” studies generally had a higher percent inclusion of the items, thereby greater transparency, compared to “pilot” studies (Table 3). Randomized studies and observational studies consistently had a higher percent inclusion of the criteria for transparency, compared to non-randomized interventional studies (Table 3). Studies collecting qualitative data were found to have lower percent inclusion for each criteria compared to quantitative studies and studies that collected both types of data (Table 3). Industry-funded studies had a lower percent inclusion rate for almost all criteria, compared to non-industry-funded studies (Table 3). Studies submitted for review prior to 2017 and after or during 2017 had comparable percent inclusion for each criterion (Table 3). Studies that stated intent to assess feasibility, specific feasibility objectives, and progression criteria in their protocols had much higher percent inclusion of the criteria for transparency compared to studies that did not (Table 3). The same trends were discovered for studies post REB review (Table 3).

**Table 3:** Relative inclusion of criteria for transparency based on each study characteristic for informed consent documents before and after research ethics board review

Study characteristic	Studies that use the term “pilot” or “feasibility” in the title of the consent forms (%)		Studies that define the term pilot/ feasibility in consent forms (%)		Studies that state objective to assess feasibility in consent forms (%)		Studies that state specific feasibility objectives in consent forms (%)	
	Before	After	Before	After	Before	After	Before	After
Term used in title of the study								
“Pilot” (*n* = 147)	78.9	82.3	9.5	12.9	32.0	34.0	16.3	16.3
“Feasibility” (*n* = 32)	87.5	87.5	6.3	6.3	75.0	75.0	28.1	28.1
Both (*n* = 5)	80.0	80.0	20.0	20.0	80.0	80.0	60.0	60.0
Study design								
Randomized (*n* = 63)	74.6	77.8	15.9	22.2	44.4	47.6	20.6	20.6
Non-randomized interventional (*n* = 54)	74.1	74.1	0.0	0	31.5	33.3	9.3	9.3
Observational (*n* = 67)	91.0	95.5	10.4	11.9	44.8	44.8	26.9	26.9
Data collected								
Quantitative (*n* = 121)	80.2	82.6	10.7	14.9	38.8	40.5	19.8	20.7
Qualitative (*n* = 12)	58.3	58.3	0.0	0	33.3	33.3	16.7	8.3
Both (*n* = 51)	86.3	90.2	7.8	7.8	47.1	49.0	19.6	19.6
Source of funding								
Industry funded (*n* = 13)	76.9	76.9	0.0	0.0	30.8	30.8	23.1	23.1
Non-industry funded (*n* = 171)	80.7	83.6	9.9	12.9	41.5	43.3	19.3	19.3
Year of submission								
2016 or prior (*n* = 135)	81.5	83.0	8.9	10.4	40.0	41.5	21.5	21.5
2017 onward (*n* = 49)	77.6	83.7	10.2	16.3	42.9	44.9	14.3	14.3
Objectives from protocol state intent to assess feasibility (*n* = 136)	80.1	82.4	11.8	15.4	54.4	56.6	26.5	26.5
Objectives from protocol do not state intent to assess feasibility (*n* = 48)	81.3	85.4	2.1	2.1	2.1	2.1	0.0	0.0
Specific feasibility objectives stated in protocol (*n* = 131)	79.4	82.4	11.5	15.3	54.2	56.5	27.5	27.5
No specific feasibility objectives stated in protocol (*n* = 53)	83.0	84.9	3.8	3.8	7.5	7.5	0.0	0.0
Progression criteria stated in protocol (*n* = 62)	85.5	87.1	17.7	22.6	53.2	56.5	27.4	27.4
No progression criteria stated in protocol (*n* = 122)	77.9	81.1	4.9	6.6	34.4	35.2	15.6	15.6

Results of the exploratory multivariable logistic regression, adjusting for various study characteristics, showed that studies that collected qualitative data were much less likely to include the terms “pilot” or “feasibility” in the titles of their informed consent documents compared to studies that collected both quantitative and qualitative data (Table 4). The odds ratio (OR) was 0.19 (95% CI 0.04, 0.82; *p* = 0.027), for originally submitted informed consent documents, and 0.13 (95% CI 0.03, 0.61; *p* = 0.010), for revised informed consent documents. The OR for all characteristics and their association with inclusion of the terms “pilot” or “feasibility” in the titles of informed consent documents can be found in Table 4.

**Table 4:** Study characteristics associated with whether consent documents state “pilot” or “feasibility” in their titles (*n* = 184)

Estimated category	Reference category (odds ratio = 1)	Originally submitted informed consent forms	Final approved informed consent forms
		Odds ratio (95% confidence interval)	Odds ratio (95% confidence interval)
2017 or more recent	2016 or prior	0.81 (0.33, 1.99)	1.11 (0.41, 2.98)
Studies labelled as feasibility studies	Studies labelled as pilot studies	1.72 (0.54, 5.43)	1.31 (0.41, 4.20)
Randomized studies	Non-randomized interventional studies	0.73 (0.26, 2.03)	0.64 (0.21, 1.90)
Observational studies	Non-randomized interventional studies	1.79 (0.63, 5.15)	1.64 (0.53, 5.12)
Quantitative studies	Studies collecting both quantitative and qualitative data	0.73 (0.26, 2.00)	0.65 (0.21, 2.01)
Qualitative studies	Studies collecting both quantitative and qualitative data	0.19 (0.04, 0.82)	0.13 (0.03, 0.61)
Industry sponsored	Non-industry sponsored studies	0.73 (0.17, 3.18)	0.66 (0.15, 2.89)
Progression criteria stated in protocol	No progression criteria stated in protocol	1.74 (0.71, 4.28)	1.53 (0.59, 3.96)

Similarly, when adjusting for study characteristics, studies labelled as “feasibility” studies and studies that stated progression criteria in their protocol were associated with stating feasibility objectives in their consent documents. “Feasibility” studies were significantly more likely than “pilot” studies to state intent to assess feasibility in their informed consent documents, with an OR of 8.09 (95% CI 3.11, 21.03; *p* < 0.001), for originally submitted informed consent documents, and an OR of 7.44 (95% CI 2.85, 19.41; *p* < 0.001), for revised informed consent documents (Table 5). Studies that stated the progression criteria in their protocols were more likely to state intent to assess feasibility in their informed consent documents, compared to studies that did not, with an OR of 2.37 (95% CI 1.14, 4.91; *p* = 0.021), for originally submitted informed consent documents, and an OR of 2.59 (95% CI 1.25, 5.36; *p* = 0.011), for revised informed consent documents (Table 5). The OR for all characteristics and their associations with stating the objective of assessing feasibility in the informed consent documents can be found in Table 5.

**Table 5:** Study characteristics associated with whether consent documents state intent to assess feasibility (*n* = 184)

Estimated category	Reference category (odds ratio = 1)	Originally submitted informed consent forms	Final approved informed consent forms
		Odds ratio (95% confidence interval)	Odds ratio (95% confidence interval)
2017 or more recent	2016 or prior	0.84 (0.38, 1.87)	0.80 (0.36, 1.78)
Studies labelled as feasibility studies	Studies labelled as pilot studies	8.09 (3.11, 21.03)	7.44 (2.85, 19.41)
Randomized studies	Non-randomized interventional studies	2.34 (0.91, 6.03)	2.49 (0.97, 6.35)
Observational studies	Non-randomized interventional studies	2.02 (0.84, 4.85)	1.83 (0.77, 4.35)
Quantitative studies	Studies collecting both quantitative and qualitative data	0.49 (0.22, 1.09)	0.47 (0.21, 1.04)
Qualitative studies	Studies collecting both quantitative and qualitative data	0.66 (0.16, 2.76)	0.63 (0.15, 2.61)
Industry sponsored	Non-industry sponsored studies	0.38 (0.09, 1.65)	0.34 (0.08, 1.46)
Progression criteria stated in protocol	No progression criteria stated in protocol	2.37 (1.14, 4.91)	2.59 (1.25, 5.36)

## Discussion

This is the first study, to our knowledge, looking at transparency of informed consent in PAFS. We found that whilst most studies reported the term “pilot” or “feasibility” in the title, less than half stated intent to assess feasibility in the informed consent documentation, and few included a definition of a pilot or feasibility study, the specific feasibility objectives, and the progression criteria in the informed consent documents. Only one study was found to include all five criteria. These results support our hypothesis that the transparency of informed consent in PAFS is, in many cases, inadequate.

Our secondary objectives included assessing whether there is a difference between the originally submitted and revised versions of the informed consent documents, with revisions suggested and made to obtain ethics approval, specifically in addressing the issues and criterion discussed in the primary objective. We found that after REB review, the proportion of studies stating “pilot” or “feasibility” in the title, the definition of a pilot or feasibility study, or the objectives to assess feasibility in the informed consent documents increased between 1.6% and 2.8%. There was no change in studies stating the specific feasibility objectives or progression criteria. This suggests that there is little improvement in transparency of informed consent in PAFS as a result of the REB review process at this center, and this is likely the case at other institutions and in other countries as well.

Taking a closer look at the first criteria, whether studies used the term “pilot” or “feasibility” in the title of the informed consent documents, we expected nearly 100% inclusion of this item, as the title of the study is to be identical to the title of the informed consent documents, according to the HiREB template for informed consent forms [21]. It is unclear why many researchers are not including the terms “pilot” or “feasibility” in the informed consent documents, when they are included in the title of the study. Perhaps researchers fear that recruitment or retention rates would drop if participants knew the project was a feasibility study, although there is no evidence to support the notion that recruitment/retention would decrease. Nonetheless, more than a quarter of PAFS lacked the terms “pilot” or “feasibility” in the title of the informed consent documents, and thus researchers and REBs should be made aware of this issue and cognisant of addressing it when designing and reviewing informed consent documents.

Most research ethics guidelines state that language used in informed consent forms should be in lay terms [11–16]. The terms “pilot study” and “feasibility study” are technical research terms that lay audiences should not be expected to understand, and yet only 12% of final informed consent documents contained some definition or explanation of what a pilot or feasibility study is. This number is likely to be similarly low across other REBs as well. The use of inappropriately complex language in consent documents is not limited to PAFS. O’Sullivan et al. evaluated the reading difficulty of consent documents for various studies and found that 91.6% of studies had ‘Fairly Difficult’ (40.3%) or ‘Difficult’ readability levels [22]. Although the issue of readability is not unique to PAFS, defining pilot or feasibility studies in their consent documents is important for transparency and needs to be addressed by REBs, researchers, and research ethics guidelines.

The TCPS2 was updated in 2018 to address informed consent in pilot studies, stating that researchers have an ethical responsibility to communicate the purpose and nature of pilot studies to participants when seeking consent [16]. However, our results indicate that most PAFS studies failed to describe their feasibility objectives, implicating that researchers and REBs are not providing adequate transparency to participants in the informed consent process of PAFS. Various research ethics guidelines state that researchers ought to explain research procedures and methods via the informed consent process, yet they do not comment on how this applies to pilot studies [11–15]. It is possible that researchers and REBs are unaware of how to ensure transparency in the informed consent process, as it pertains to pilot studies. Research ethics guidelines should address this issue by specifically addressing informed consent in pilot studies with clear descriptions on what items should be communicated in the informed consent documents. Moreover, training for researchers and REBs should include guidance on informed consent in pilot studies, and resources, checklists, and templates should be developed and used in the design and review of informed consent documents for pilot studies.

Left unaddressed, this issue of inadequate transparency of informed consent in pilot studies has severe ethical implications. If participants are left unaware of the purpose of the studies they volunteer in, their rights can be violated, the researchers’ credibility damaged, and trust between participants and researchers broken. If participants learn they were misinformed during the informed consent process they could feel betrayed; question the integrity of researchers; decide not to participate in future studies; question the integrity of research and evidenced based medicine; and even take legal action against researchers, research sponsors, or research ethics committees. This is especially concerning in the “post-truth society” described by Iyengar and Massey [23], in which scientists regularly encounter targeted media and social media campaigns of fake news, misinformation, and disinformation. Thus, it is imperative that the issue of informed consent in PAFS is addressed urgently.

### Study characteristics associated with transparency

Our study also aimed to determine methodological characteristics associated with reporting or inclusion of the five criteria for transparency in informed consent documents. Due to the few number of studies including each criteria for transparency, we were only able to perform the binary logistic regression for studies that incorporated the terms “pilot” or “feasibility” in their title and stated the primary objectives of the study were to assess feasibility in the informed consent documents.

Studies that collected qualitative data were significantly less likely to included “pilot” or “feasibility” in the title of the informed consent documents, compared to studies that collected both qualitative and quantitative data. It is unclear as to why this correlation exists and what impact it has, if any, on the design or development of pilot studies and informed consent documents.

Our regression analysis showed that “feasibility” studies were significantly more likely to state intent to assess feasibility in their consent documents than “pilot” studies. This could be a reflection of how REBs focus their attention and suggested revisions. Perhaps the term “feasibility study” is more closely associated with assessment of feasibility compared to the term “pilot study,” and thus it leads to more suggested revisions to state the feasibility objectives in the informed consent documents.

We also found that studies that stated the progression criteria for the study to lead to a larger study in their protocols were more likely to state intent to assess feasibility in their informed consent documents, compared to studies that did not state these criteria. This suggests that well planned PAFS tend to be more transparent in their informed consent practices, by stating their objectives are to assess feasibility in their informed consent documents.

An interesting trend, although not statistically significant, is that industry sponsored studies were less likely to include “pilot” or “feasibility” in the title of their informed consent documents. They were also less likely to state that the primary objectives of the study were to assess feasibility in the informed consent documents. Although the finding is not statistically significant, it suggests that industry-funded studies are less likely to have transparent informed consent practices for PAFS. Perhaps REBs should pay special attention when revising informed consent documents of industry-funded PAFS.

With respect to temporality, the CONSORT extension [20] for pilot studies was published in late 2016 and for this reason we categorized the study dates to before 2017 and after or during 2017. We expected the transparency of informed consent would increase after 2016, due to the publication of recommendations in the CONSORT extension. However, we found the opposite trend was true and that a fewer proportion of studies communicated their objectives were to assess feasibility in the informed consent documents. It appears that the CONSORT extension has not yet improved communication of the feasibility nature of the objectives of PAFS, again highlighting the need for more resources and guidance for researchers and REBs in addressing the lack of transparency of informed consent in PAFS.

### Limitations

It is important to address the limitations of this study, as it only looked at one REB, in Canada. The results may not be representative of all REBs; however, they are likely similar to other REBs. Our analysis was also limited by the nature of the data. We were powered for our primary objective but to address some secondary objectives our data only allowed us to conduct multivariable analysis for two of our five primary outcome measures (whether the consent documents state “pilot” or “feasibility” in their titles and whether they state intent to assess feasibility). The other three outcomes did not have enough events in the response variable for the number of independent variables that we were fitting in the model. Thus, these models would create over fitting and be unstable. Nonetheless, this study provides empirical evidence that informed consent in PAFS lacks transparency and that this issue needs to be addressed. Future research should focus on quantifying this issue at other centers and in other countries, identifying reasons for poor informed consent practices, and developing ways to improve informed consent in PAFS.

## Conclusion

Informed consent in pilot studies submitted to the HiREB is not transparent. This is a serious concern that can lead to severe consequences and ethical implications. Although steps have been taken, like the CONSORT extension and TCPS2 update addressing pilot studies, this is insufficient. More research ethics guidelines need to address informed consent in PAFS and in more depth, that provides clear and comprehensive instruction. Researchers and REBs need to be made aware of issues of informed consent in PAFS. Tools and resources should be developed on how to appropriately address informed consent in PAFS to uphold the rights of participants.

## Supplementary Information

**Additional file 1: Table 1A.** Percentage of studies that assess feasibility, state specific feasibility objectives and progression criteria (*n*=184). **Table 1B.** studies’ specific feasibility objectives (*n*=184)

**Additional file 2: Table 1.** Characteristics of studies that clearly communicate each criteria of transparency before and after research ethics board review

## Data Availability

The datasets used in this study are available from the corresponding author upon request. They encourage collaborations.

## Abbreviations

PAFS: Pilot and feasibility studies
REB: Research ethics board
TCPS2: Tri-Council Policy Statement 2
HiREB: Hamilton integrated Research Ethics Board
CI: Confidence interval
CONSORT: Consolidated Standards of Reporting Trials
