# Supplementary material for: Transparency of informed consent in pilot and feasibility studies is inadequate: a single-center quality assurance study
Source: Pilot Feasibility Stud. 2021 Apr 16;7:96. doi: 10.1186/s40814-021-00828-w (PMC8051114; doi:10.1186/s40814-021-00828-w)
Supplement: Supplementary file 1 — Additional file 1: Table 1A. Percentage of studies that assess feasibility, state specific feasibility objectives and progression criteria (n=184). Table 1B. studies’ specific feasibility objectives (n=184) [file 40814_2021_828_MOESM1_ESM.docx]

Appendix 1

Appendix 1A

Table 1A: Percentage of studies that assess feasibility, state specific feasibility objectives and progression criteria (n=184)

| **Study protocols clearly state:** | **Percent of studies (%) (95% Confidence Interval)** |
| --- | --- |
| Objective of assessing feasibility | 73.9 (67.6, 80.3) |
| Specific feasibility objectives | 71.2 (64.7, 77.7) |
| Progression criteria | 33.7 (26.9, 40.5) |

Appendix 1B

Table 1B: studies’ specific feasibility objectives (n=184)

| **Specific feasibility objectives** | **Percent of studies (%) (95% Confidence Interval)** |
| --- | --- |
| None | 28.8 (22.3, 35.3) |
| Process | 70.7 (64.1, 77.2) |
| Resources | 21.2 (15.3, 27.1) |
| Management | 9.2 (5.1, 13.4) |
