# Supplementary material for: Transparency of informed consent in pilot and feasibility studies is inadequate: a single-center quality assurance study
Source: Pilot Feasibility Stud. 2021 Apr 16;7:96. doi: 10.1186/s40814-021-00828-w (PMC8051114; doi:10.1186/s40814-021-00828-w)
Supplement: Supplementary file 2 — Additional file 2: Table 1. Characteristics of studies that clearly communicate each criteria of transparency before and after research ethics board review [file 40814_2021_828_MOESM2_ESM.docx]

Appendix 2

Table 1: Characteristics of studies that clearly communicate each criteria of transparency before and after research ethics board review

| **Study characteristic** | **Studies that use the term “pilot” or “feasibility” in the title of the consent forms (%)** | | **Studies that define the term pilot/ feasibility in consent forms (%)** | | **Studies that state objective to assess feasibility in consent forms (%)** | | **Studies that state specific feasibility objectives in consent forms (%)** | |
| --- | --- | --- | --- | --- | --- | --- | --- | --- |
|  | **Before (n=148)** | **After (n=153)** | **Before (n=17)** | **After (n=22)** | **Before (n=75)** | **After (n=78)** | **Before (n=36)** | **After (n=36)** |
| **Term used in title of the study** | | | | | | | | |
| **“Pilot”**  **“Feasibility”**  **Both** | **78.4**  **18.9**  **2.7** | **79.1**  **18.3**  **2.6** | **82.4**  **11.8**  **5.9** | **86.4**  **9.1**  **4.5** | **62.7**  **32.0**  **5.3** | **64.1**  **30.8**  **5.1** | **66.7**  **25.0**  **8.3** | **66.7**  **25.0**  **8.3** |
| **Study design** | | | | | | | | |
| **Randomized**  **Non-randomized interventional**  **Observational** | **31.8**  **27.0**  **41.2** | **32.0**  **26.1**  **41.8** | **58.8**  **0.0**  **41.2** | **63.6**  **0.0**  **36.4** | **37.3**  **22.7**  **40.0** | **38.5**  **23.1**  **38.5** | **36.1**  **13.9**  **50.0** | **36.1**  **13.9**  **50.0** |
| **Data collected** | | | | | | | | |
| **Quantitative**  **Qualitative**  **Both** | **65.5**  **4.7**  **29.7** | **65.4**  **4.6**  **30.1** | **76.5**  **0.0**  **23.5** | **81.8**  **0.0**  **18.2** | **62.7**  **5.3**  **32.0** | **62.8**  **5.1**  **32.1** | **66.7**  **5.6**  **27.8** | **69.4**  **2.8**  **27.8** |
| **Year of submission** | | | | | | | | |
| **2016 or prior**  **2017 onward** | **74.3**  **25.7** | **73.2**  **26.8** | **70.6**  **29.4** | **63.6**  **36.4** | **72.0**  **28.0** | **71.8**  **28.2** | **80.6**  **19.4** | **80.6**  **19.4** |
| **Desired sample size**  **Median (min, max)** | **50 (5, 1152)** | **50 (5, 1152)** | **70 (5, 400)** | **65 (5,400)** | **42 (5, 384)** | **46 (5, 384)** | **40 (5, 384)** | **41 (5, 384)** |
| **Industry funded** | **6.8** | **6.5** | **0.0** | **0.0** | **5.3** | **5.1** | **8.3** | **8.3** |
| **Objectives state intent to assess feasibility** | **73.6** | **73.2** | **94.1** | **95.5** | **98.7** | **98.7** | **100** | **100** |
| **Specific feasibility objectives stated** | **70.3** | **70.6** | **88.2** | **90.9** | **94.7** | **94.9** | **100** | **100** |
| **Progression criteria stated** | **35.8** | **35.3** | **64.7** | **63.6** | **44.0** | **44.9** | **47.2** | **47.2** |
